# Gene network analyses point to the importance of human tissue kallikreins in melanoma progression

**DOI:** 10.1186/1755-8794-4-76

**Published:** 2011-10-27

**Authors:** Waleska K Martins, Gustavo H Esteves, Otávio M Almeida, Gisele G Rezze, Gilles Landman, Sarah M Marques, Alex F Carvalho, Luiz F L Reis, João P Duprat, Beatriz S Stolf

**Affiliations:** 1 Hospital A.C. Camargo, São Paulo, Brazil; 2 Ludwig Institute for Cancer Research, São Paulo, Brazil; 3 Instituto de Matemática e Estatística, Universidade de São Paulo, Brazil; 4 Instituto de Ciências Biomédicas, Universidade de São Paulo, Brazil; 5 Instituto de Química, Universidade de São Paulo, Brazil; 6 Centro de Ciências e Tecnologia da Universidade Estadual da Paraíba, Paraíba, Brazil; 7 Hospital Sírio-Libanês, São Paulo, Brazil

## Abstract

**Background:**

A wide variety of high-throughput microarray platforms have been used to identify molecular targets associated with biological and clinical tumor phenotypes by comparing samples representing distinct pathological states.

**Methods:**

The gene expression profiles of human cutaneous melanomas were determined by cDNA microarray analysis. Next, a robust analysis to determine functional classifications and make predictions based on data-oriented hypotheses was performed. Relevant networks that may be implicated in melanoma progression were also considered.

**Results:**

In this study we aimed to analyze coordinated gene expression changes to find molecular pathways involved in melanoma progression. To achieve this goal, ontologically-linked modules with coordinated expression changes in melanoma samples were identified. With this approach, we detected several gene networks related to different modules that were induced or repressed during melanoma progression. Among them we observed high coordinated expression levels of genes involved in a) cell communication (*KRT4*, *VWF *and *COMP*); b) epidermal development (*KLK7*, *LAMA3 *and *EVPL*); and c) functionally related to kallikreins (*EVPL*, *KLK6, KLK7, KLK8*, *SERPINB13*, *SERPING1 *and *SLPI*). Our data also indicated that hKLK7 protein expression was significantly associated with good prognosis and survival.

**Conclusions:**

Our findings, derived from a different type of analysis of microarray data, highlight the importance of analyzing coordinated gene expression to find molecular pathways involved in melanoma progression.

## Background

Cutaneous melanoma is considered a complex multigenic and multifactorial disease that involves both environmental and genetic factors [1-3]. Its tumorigenesis is often explained as a progressive transformation of normal melanocytes to nevi that subsequently develop into primary cutaneous melanomas (PCM) [4,5]. In fact, the development of PCM is a classical example of a neoplasm progression through discrete stages with well-known clinical and histological features. In spite of its obvious oversimplification, this model incorporates the notion that signals triggered by cell-cell and cell-extracellular matrix interactions are critical for maintaining melanocyte homeostasis [6]. Deregulation of these interactions may disturb the equilibrium of epidermal melanin units a leading to continuous proliferation of melanocytes and development of PCM. In fact, the loss of intercellular adhesion plays a critical role as a limiting factor during the early steps of melanoma invasion into the dermis, tumor dissemination and metastasis [7,8]. There are still several gaps in the exact cellular mechanisms that occur between PCM at early stages (I or II) and its potential to disseminate as late stage metastatic melanoma (MM). In an effort to clarify this issue, a wide variety of high-throughput microarray platforms have been applied [9,10].

Microarray studies on human skin melanoma produced a plethora of data without resulting in breakthrough in melanoma diagnosis or management of melanoma patients [9]. There remains considerable debate in the literature as to why comparative microarray studies do not completely coincide with each other [10]. Some reasonable explanations are that the studies differ in sample classification (subdivisions), cohort size, type of array and statistical analysis used. These differences make comparisons quite difficult and result in a reduced total cohort size and diversity, since independent cohorts from different studies are hard to be summed [9].

All the studies performed to date analyzed differences between melanoma cell lines [11,12], between melanocytes and PCMs [13], PCMs from different stages and/or PCMs and metastatic tumors [10,14-19]. In all these reports, the genes differentially expressed between each two sample types were identified independently, and in some studies were classified according to GO groups. The GOs most frequently altered according to tumor thickness and/or metastasis were cell proliferation, cell cycle regulation and mitosis, cell-cell adhesion and cell extracellular matrix interaction [18]. Interestingly, many genes up or downregulated in thick compared to thin PCMs maintained the same profile in metastatic tumors, indicating that thick primary melanoma tumors may have the gene expression signature of MM tumors [9,10]. Together, these studies identified several transcriptional differences that provide insights into the progression of melanoma and the metastatic process. However, to delineate the main differences between the molecular signatures of PCM and MM samples, they have focused only on the differential expression profiles based on multiple hypothesis testing such as Wilcoxon test [19], Student's t-test [17] or significance analysis of microarrays (SAM) [9,15,18].

In the present study we adopted a different strategy: we searched for genes whose expression was coordinately altered between two conditions: PCMs with different Breslow's thickness index or PCMs versus metastasis. This recently described approach looks for changes in the expression of co-regulated genes that share a common function. Rather than single-gene analysis, this strategy might predict whole pathways that are involved in a particular biological process, named biological module. This concept was initially described by Segal and collaborators [20] and was successfully applied by our group to study esophagus and stomach cancers [21].

Using this approach we identified groups of melanomas in which different meaningful ontologically-linked modules were induced or repressed. We observed reduced expression of genes involved in cell communication, epidermal development, skin desquamation and cell adhesion in MM samples. This loss of cell-stromal interactions may reflect the gain of migratory potential of the metastatic cell type. Additionally, we performed comprehensive pair-wise comparisons among genes within each module and determined their linear correlation coefficients. We identified linked changes in the expression of human tissue kallikreins (hKLKs), a family of serine-proteases that are drawing increased attention due to their association with various forms of cancer and other diseases [22-29]. We showed a coordinated decrease in the expression of hKLKs 6, 7, 8 and 13 (in terms of mRNA and protein) as the metastatic signature emerges, suggesting a role of these proteases in the epithelial-mesenchymal transition of PCM cells. This also confirmed previous observations that the expression of hKLK7 is significantly associated with good prognosis and survival [16,30].

hKLKs are located in a cluster on region q13.3-13.4 of chromosome 19 [22,28,31-33] and have been implicated in carcinogenesis due to their role as biomarkers for the screening, diagnosis, prognosis, and monitoring of various cancers including prostate, ovarian, breast, testicular, lung and cervical [22-29]. hKLKs catalyze the degradation of intercellular cohesive structures (corneodesmosomes) at skin surface, being expressed in the upper spinous and granular layers of the epidermis and playing a crucial role during keratinocyte cell shedding - i.e. desquamation [27,32-34].

Many functional aspects of hKLKs need to be better explored, including their regulation, identification of specific substrates, their participation in proteolytic cascades, and their clinical applicability as cancer biomarkers and therapeutic targets. Our data suggest that the clinical value of several hKLKs (6, 7, 8 and 13) as biomarkers for human cutaneous melanoma should be further evaluated.

## Methods

### Patients and experimental samples

Tissue samples representing independent PCM and MM samples were obtained from Hospital A. C. Camargo. All patients signed an informed consent, and the study was approved by the internal ethics committee of the Hospital A. C. Camargo/Ludwig Institute for Cancer Research. Tissue samples obtained by surgery were snap-frozen in liquid nitrogen right after removal and biopsy samples were immediately collected in RNAlater™ (Ambion, Austin, TX). Parallel fragments of the tissue sections were embebed in paraffin and stained with hematoxylin/eosin for confirmation of diagnosis. All samples were analyzed, dissected and re-analyzed by an expert pathologist (Dr. Gilles Landman). Non-tumor areas were removed from frozen samples by dissection and only samples containing at least 70% of tumor cells within their stroma were selected for RNA extraction. A detailed description of the 60 samples is presented in additional file 1.

### Reference sample

Reference sample was obtained by combining five human cutaneous melanoma cell lines: SK-MEL 05; 17; 28; 37 and 188, kindly provided by Dr. Alan Houghton (MSKCC, New York, NY, USA). The cells were cultured in Dulbecco Modified Eagle Medium (Sigma Chemical Co., St. Louis, MO) supplemented with 10% (v/v) fetal calf serum, 100 U/mL of penicillin, and 100ρg/mL of streptomycin. Cell culture was maintained in a 37°C incubator at a moist atmosphere of 5% carbon dioxide.

### RNA extraction, amplification, cDNA labeling, and hybridization

Total RNA from tumor specimens and melanoma cell lines were isolated with RNeasy^® ^Midi-columns (Qiagen, Valencia, CA) following the manufacturer's instructions. The quality of total RNA was evaluated by agarose gel electrophoresis and by measuring absorbance ratios of 260/280 and 260/230. 3.0 μg of total RNA was linearly amplified using T7-based *in vitro *transcription following an optimized protocol [35]. The quality of the amplified mRNA was evaluated by the same methods employed for total RNA. Amplified 5' Poly (U) antisense RNA was converted into cDNA in the presence of aminoallyl-modified dUTP and coupled to N-hydroxysuccinimidyl esters of Cy3 and Cy5 (Molecular Probes^®^, Invitrogen, Carlsbad, CA) [36]. Pre-hybridization, hybridization and washing steps were performed as described previously [21], and slides were scanned on a laser scanner (ScanArray Express, Perkin Elmer Life Sciences, Boston, MA). Data were extracted with ScanArray Express software (Perkin Elmer Life Sciences, Boston, MA) using the histogram method. The raw data from hybridizations and experimental conditions are available on the Gene Expression Omnibus website, accession number GSE17275.

### Microarray platform

We employed a customized microarray platform that contains 4,608 cDNAs representing unique genes with known full-length sequences that were selected from the ORESTES [37,38] clone collection derived from the FAPESP/LICR Human Cancer Genome Project. A map and gene list for the described microarray platform is available at Gene Expression Omnibus website, accession number GPL1930.

### Dual-label design

A dual-labeling system was used to generate replicate data while controlling for possible differences in the efficiency of dye incorporation by different mRNA samples. Two mRNA samples (experimental and reference) were separately reverse-transcribed, labeled, mixed, and hybridized in duplicate, with the dye assignments reversed in the second hybridization. Reference sample was included in all glass slides to enable the calculation of relative expression measures for all samples. Since this reference should not contrast to the experimental samples [39], we prepared it from a pool of equal amounts of total RNA from five human cutaneous melanoma cell lines, as cited above.

### Statistical analysis

Data analysis was done using R, an open source interpreted computer language for statistical computation and graphics [40,41] obtained from the Contributed R Archive Network http://www.rproject.org, and tools from the Bioconductor Project http://www.bioconductor.org. After image acquisition and quantification, spots with signals lower or equal to background were excluded from the normalization step. Normalization was performed using LOWESS, a local non-linear regression fitting method, using span 0.4 and degree 1 for local-background subtracted spots [21]. Pearson's correlation was used to analyze correlations between experimental and replicate data.

The survival endpoint was defined as the time interval from diagnosis until death, and was used as an indicator of the clinical outcome. Surviving patients and patients who had died from melanoma were censored at the date of the last follow-up and at the date of death, respectively. Patients whose death was not related to cancer were censored at the date of death.

To identify sequences associated with reduced rates of death from melanoma, we analyzed data for 45 of the 60 patients who had survived for at least 4 years of follow-up. Four patients who had died from unrelated causes and 11 patients without a reported event but with a follow-up of less than 4 years were excluded from this analysis. Patients were then separated into a group of 19 patients who had survived for 4 or more years, and a group of 26 patients who had died within 4 years of diagnosis.

### Ontologically-linked gene sets

To search for gene expression profiles that were associated with specific groups of melanomas but not necessarily related to an individual sample, we applied a module map similar to that described by Segal et al. [20]. This analysis evaluated the differential induction or repression of gene networks representing biological modules. These modules were defined using a robust analysis to determine functional classifications and make predictions based on data-oriented hypotheses using mathematical tools, as already described by our group [42].

We selected biologically meaningful gene sets belonging to the same functional category or pathway related to biological modules that are important for melanoma progression such as apoptosis, cell signaling pathways, cell adhesion, cell communication, cell differentiation, cell cycle progression, epidermal development, keratinocyte differentiation, neuronal phenotype, cell shedding, invasion, metastasis and others. After searching the GeneCards^® ^Human Gene Database [43], Gene Decks Assorted Gene Sets (GDAGS) and Gene Ontology Hierarchy [44] for the genes mentioned above, we compiled 4,523, 1,225 and 3,298 genes, respectively. Of those 4,523 genes, 1,306 were represented in our ORESTES platform and were organized into 123 modules according to the gene annotation shown in GeneCards^®^. Furthermore, 69 tissue or function-specific expressed genes obtained from the available literature were fitted into the metastasis signature [11,15,45-47] and functionally hKLK-related genes [31-33,48,49].

### Identification of samples with significant changes in the status of modules

After data normalization, individual gene expression values were standardized by subtracting the average expression calculated over all the samples. This process adjusted the mean values of each gene to zero and generated a discrete version of the expression values, where each gene in a sample was defined as 1 (induction) if its expression was at least 2-fold greater than the average, as -1 (repression), if its expression value was reduced by at least 2-fold or as 0 (equal expression).

The number of induced and repressed genes in a module of interest was counted for each melanoma sample. Next, to assess the significance (p-value lower than 0.05) of the fraction of induced and repressed genes identified for each sample we applied probabilistic hypotheses tests following hypergeometric distribution with known parameters [20]. After defining the status of a given module (as induced or repressed) in each melanoma sample, we counted the number of induced or repressed samples that comprised a melanoma set discriminated according to pathological or clinical tumor characteristics: tumor subtype (PCM and MM samples); melanoma progression (T1+T2, T3, T4 and MM samples); cancer stage (I, II, III and IV), metastasis site (LN, CUT and VISC) and clinical outcome (Surviving and Death). Again using a hypergeometric distribution, we classified each module as significantly induced or repressed across the groups of interest (using p-value lower than 0.05).

### Testing consistency of a gene with the status of a module

For any gene (G) belonging to a given module in the wider gene expression dataset, Segal et al. [20] proposed a score that described whether the expression of G was consistent with the overall changes in the expression of the module. We first identified the subsets of samples in which G was significantly induced (*I*) or repressed (*R*). Next, we measured the extent to which the expression of *G *changed by approximately 2-fold in samples *I *(or *R*); p^i^_*s*_ and p^r^_*s*_ denote the fraction of genes in sample (*S*) that are induced or repressed, respectively. The score of *G *is the sum, over all samples, of weights that depend on the status of *S *itself and the status of *G *in that sample. An induced *S *in which *G *is also induced contributes with weight -log (p^i^_*s*_), while a repressed *S *in which *G *is also repressed contributes with weight -log (p^r^_*s*_). Consequently, a given *G *with high score is a gene that behaves consistently with respect to the status of the module. The statistical significance of a high score may be easily computed since, under the null hypothesis (genes in each sample are equally likely to be induced or repressed), the score is the sum of independent Bernoulli random variables [20,42].

### Discrimination of melanoma samples using SOM

After definition of the genes with higher scores (as described above), we used a list of 44 non-redundant genes to discriminate melanomas samples into two main groups, using Self-Organizing Maps (SOM). Once clusters were obtained, samples were organized hierarchically based on their correlation distances. Next, we used Chi-square and Mann-Whitney tests to assess differences between the SOM groups according to the clinical and pathological variables of each sample.

### Relevance networks

To identify genes with coordinated expression profiles we made comprehensive pair-wise comparisons of genes within each module and determined their correlation coefficients. This is known as a relevance network and was originally proposed by Butte et al. [50]. This method computes the squared linear Pearson correlation coefficient$r_{ij}^{2}$ between all gene pairs for each melanoma set (defined in the section *"Identifying samples with significant changes in the status of modules"*), defining a fully connected graph. Using a re-sampling method, the algorithm estimates a cut-off value *c *and splits the graph into small sub-graphs where $r_{ij}^{2}>c$. These sub-graphs are the relevance networks (RNs).

We adapted this method to identify significant differences between two RNs, as described previously [51]. The linear Pearson correlation coefficients of two sample subtypes $r_{ij}^{1}$ were computed between every pair of genes. A Fisher's Z-transformation [52] was used to translate the probability distribution of the random variable $r_{ij}^{1}-r_{ij}^{2}$into an approximated standard normally distributed random variable, permitting the identification of pairs of genes with significant (positive or negative) differences between $r_{ij}^{1}$ and $r_{ij}^{2}$ (p-value lower than 0.05).

Based on the p-value of the previous test, we then constructed a matrix using a green to red heat map scale, where green represents a correlation of -1, red represents a correlation of +1, and black indicates an absence of correlation.

### Immunohistochemistry staining

To validate the correlation pattern between *KLK6 *and *KLK7 *we used a tissue microarray (TMA) platform containing 162 independent PCM and 28 MM samples elaborated by Rezze et al. [30]. hKLK6 (goat anti-kallikrein 6, polyclonal, R&D Systems, Minneapolis, MN) and hKLK7 (goat anti-kallikrein 7, polyclonal, R&D Systems, Minneapolis, MN) were used in a standard labeling protocol. Briefly, sections were deparaffinized in xylene and rehydrated with graded alcohol and tap water. Antigen retrieval was done in a heat-controlled pressure cooker (PASCAL pressure cooker, Dako, Carpinteria, CA) containing 10 mM sodium citrate buffer at pH 6.0 (Dako, Carpinteria, CA). Endogenous peroxidase activity was quenched with 3.0% (v/v) hydrogen peroxide (Dako, Carpinteria, CA) in methanol for 15 minutes. Protein block (Dako, Carpinteria, CA) was applied for 20 minutes at room temperature in a humidified chamber. Primary antibodies were incubated for two hours at room temperature in a humidified chamber according to pre-established dilutions for each antibody. The LSAB Visualization System (Dako, Carpinteria, CA) and 3,3-diaminobenzidine chromogen (Dako, Carpinteria, CA) were used according to the manufacturer's instructions. After dehydration, glass slides were coated and mounted with Entellan mounting medium (Merck KGaA, Darmstadt, Germany).

TMA samples were analyzed with the ACIS III (Automated Cellular Imaging System-ChromaVision Medical Systems^®^, San Juan Capistrano, CA) digital microscopy system, according to Rezze et al. [30].

Comparative statistical analysis was used to characterize hKLKs expression according to pathological and clinical tumor characteristics. Kolmogorov-Smirnov test indicated that the population did not have a normal distribution. Hence, Mann-Whitney and Kruskal-Wallis non-parametric tests were used to compare median protein expression values between two and three groups, respectively. The correlations between hKLKs markers were examined using Spearman's coefficient. P-values lower than 0.05 were considered significant.

## Results

### Evaluation of multiple gene expression profiles by ontologically-oriented analysis

In order to identify ontologically-linked gene sets with coordinated expression changes that correlate with melanoma progression we analyzed the expression profiles of 60 independent tumors representing PCMs (20) and MMs (40) (see additional file 1). We used a customized microarray platform containing 4, 608 ORESTES that has been successfully used in other studies of our group [53].

All genes represented in the ORESTES microarray platform were grouped according to biological process as described by public databases GeneCards^® ^[43] and Gene ontology [44]. Furthermore, we grouped tissue or function-specific genes according to the available literature.

In the first-pass analysis we identified sample groups with significant changes in the status of each module. The categories and the sample groups considered were: tumor subtype (PCM and MM samples); melanoma progression (T1+T2, T3, T4 and MM samples); cancer stage (I, II, III and IV), metastasis site (LN, CUT and VISC) and clinical outcome (Surviving and Death). By this analysis we identified 22 significantly non-redundant modules that were repressed or induced according to the categories (Table 1).

**Table 1:** The most altered modules based on all comparisons.

MODULES	TUMOR SUBTYPE		MELANOMA PROGRESSION				**CANCER STAGE**^b^				METASTASIS SITE			CLINICAL OUTCOME^c^	
	
	PCM	MM	T1+T2^a^	T3^a^	T4^a^	MM	I	II	III	IV	LN	CUT	VISC	S	D
*Cell communication (GD)*	*0.5*	*-0.4*	*0.0*	*0.0*	*0.0*	*-0.4*	*0.0*	*0.0*	*0.0*	*-0.4*	*0.0*	*-0.4*	*0.0*	*---*	*---*
*Functionally hKLK-related genes*	*0.5*	*-0.2*	*0.0*	*0.7*	*0.0*	*-0.2*	*0.0*	*0.6*	*0.0*	*0.0*	*---*	*---*	*---*	*---*	*---*
*Epidermal development (GO8544)*	*0.5*	*-0.2*	*0.0*	*0.0*	*0.0*	*-0.2*	*0.0*	*0.5*	*0.0*	*-0.3*	*---*	*---*	*---*	*---*	*---*
Peptidase activity (GO8233)	0.3	0.0	0.7	0.0	0.0	0.0	---	---	---	---	---	---	---	---	---
Neuroactive ligand receptor (GD)	0.2	0.0	0.0	0.5	0.0	0.0	---	---	---	---	---	---	---	---	---
Metastasis signature	0.0	-0.4	0.0	0.0	0.0	-0.4	0.0	0.0	0.0	-0.5	---	---	---	---	---
Cell adhesion (GO7155)	0.0	-0.3	0.0	0.0	0.0	-0.3	---	---	---	---	---	---	---	---	---
Prostaglandin and leukotriene metabolism (GD)	---	---	0.0	-0.3	0.0	0.0	---	---	---	---	---	---	---	---	---
Intercellular junction (GO5911)	---	---	0.7	0.0	0.0	0.0	---	---	---	---	---	---	---	---	---
Nervous system development (GO7399)	---	---	0.7	0.0	0.0	0.0	---	---	---	---	---	---	---	---	---
Negative regulation of progression through cell cycle (GO45786)	---	---	---	---	---	---	0.0	0.0	0.0	-0.2	---	---	---	---	---
Inflammatory response (GO6954)	---	---	---	---	---	---	0.0	0.0	0.2	0.0	---	---	---	---	---
Hydrolase activity (GO16787)	---	---	---	---	---	---	0.0	0.3	0.0	0.0	---	---	---	---	---
Extracellular region (GO5576)	---	---	---	---	---	---	0.0	0.0	0.0	-0.5	---	---	---	---	---
Extracellular matrix sensu Metazoa (GO5578)	---	---	---	---	---	---	0.0	0.0	0.0	-0.4	---	---	---	---	---
Cell motility (GO6928)	---	---	---	---	---	---	0.0	0.0	0.0	-0.2	---	---	---	---	---
Alzheimers disease (GD)	---	---	---	---	---	---	0.0	0.0	0.0	0.2	---	---	---	---	---
G Protein signaling (GO7187)	---	---	---	---	---	---	---	---	---	---	-0.2	0.0	0.0	---	---
Leukocyte transendothelial migration	---	---	---	---	---	---	---	---	---	---	0.2	0.0	0.0	---	---
Integral to plasma membrane (GO5887)	---	---	---	---	---	---	---	---	---	---	0.3	0.0	0.0	---	---
Antigen processing and presentation (GD)	---	---	---	---	---	---	---	---	---	---	0.3	0.0	0.0	---	---
Response to stress (GO6950)	---	---	---	---	---	---	---	---	---	---	0.0	0.0	-0.7	-0.4	0.0

The modules are listed by values representing their status (induced or repressed). Induced modules are depicted by positive values and repressed modules by negative values.

The modules in italic were selected for showing changes in at least four different groups in distinct category types.

^a^Breslow's thickness index according to AJCC staging system. ^b^Cancer stage according to the AJCC staging system. ^c ^Follow up set as more than 4 years: S = surviving, D = death.

Next, we evaluated the consistency of the genes that contributed most significantly (p-values lower than 0.05) to the changes described in Table 1, as already proposed by Segal et al. [20] (see additional file 2). As can be observed, the expression profiles of some genes were consistently and significantly (cut off set as 10^-5^) related to the status of tumor characteristics. Some examples are *BST2, C1QR1, CCL18, CD53, COL3A1, CTGF, FBN1, GPR56, LAMA4, LUM *and *POSTN*.

**Figure 1:**
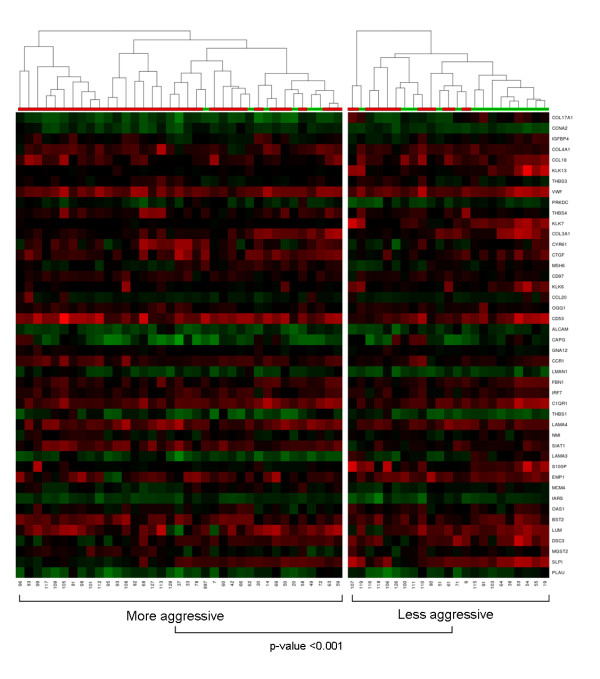
**Hierarchical representation of self-organizing maps (SOM) clustered samples based on the expression profiles of biologically meaningful modules**. The clustering of PCM and MM samples was based on similarities in the expression profiles of 44 non-redundant genes that contributed most significantly (cut off set as 10^-3.3^) to the change in the status of all 22 modules identified in this study. The SOM heatmap shows PCM samples by green bars and MM samples by red bars. Red: higher expression in the tumor than the reference mRNA; green: lower expression than the reference; black: no change compared to the reference.

A self-organizing map was then used to group melanoma samples into two major clusters on the basis of the expression profile of 44 non-redundant genes that contributed most significantly (cut off set as 10^-3.3^) to the change in the status of all modules identified here. As can be observed in Figure 1, PCM and MM samples could not be precisely clustered. However, when patient and tumor characteristics were evaluated, the samples could be separated into two distinct main groups with significant association (chi-square test) with tumor subtype (p-value < 0.001) and cancer stage (p-value = 0.003). The right cluster, identified as "less aggressive" in Figure 1, contained samples from 23 patients, comprising 14 of 20 PCM samples representing 2 stage I, 7 of 8 stage II, 4 of 19 stage III and 1 of 31 stage IV patients. The left cluster, identified as "more aggressive", contained samples from 37 patients, comprising 31 of 40 MM samples representing 8 of 19 stage III and 23 of 31 stage IV patients. The PCM (7 of 20) set clustered with most of the MM samples (left cluster) showed a significant increase (p-value = 0.019) in mean thickness compared to that of the right cluster (14.51 mm versus 3.84 mm).

### Relevance networks in human melanomas

To identify a relevance network (RN) for each subgroup defined above, a squared linear Pearson correlation coefficient was applied to determine linked expression profiles between all gene pairs within a given module. We applied this method for all modules, therefore several RNs were found. For further analysis, we considered only modules (highlighted in Table 1) that were significantly changed in at least four different groups in distinct category types.

Next, the significance of differences between two RNs was computed for two distinct conditions. Several evaluations had regular |0.4 < r < 0.6 | and moderate |0.6 < r < 0.8 | correlation coefficients with statistical significance (p-value < 0.005). However, only high correlation coefficients |0.8 < r < 1.0 | with elevated significance (cut off set as 10^-4.3^) were considered (see additional file 3).

For tumor subtype category (PCM and MM), this approach allowed us to identify a degree of correlation between the expression levels of genes involved in a) cell communication (*KRT4*, *VWF *and *COMP*); b) epidermal development (*KLK7*, *LAMA3 *and *EVPL*); and c) functionally related to kallikreins (*EVPL, KLK6, KLK7, KLK8, SERPINB13*, *SERPING1 *and *SLPI*).

No relevant networks were found with respect to metastasis site or clinical outcome (data not shown). Only three networks correlated to cancer stage, two of them in the module cell communication and one in the set of hKLK related genes. The network between two genes involved in cell connecting interactions (*GJB1 *and *DES*) showed a negative and higher correlation (r = -0.943) in stage II melanoma patients when compared to stage III melanoma patients (r = 0.54). In contrast, stage II melanoma patients showed a positive and higher correlation coefficient (r = 0.91) between serpin peptidase inhibitors (*SERPINB2 *and *SERPINB6*) in comparison to stage IV melanoma patients (r = -0.61) (see additional file 3).

For melanoma progression category (T3, T4 and MM), only genes ontologically-related to hKLKs (*EVPL, KLK6, KLK7, KLK8, KLK13 *and *SERPINB13*) showed a positive and high correlation coefficient (mean of 0.971) in T4 thick melanomas (depth > 4.0 mm), but not in metastatic tumors (MM). This suggests that the expression of hKLKs at mRNA level is associated to melanoma progression before the establishment of distant metastasis.

## Kallikreins expression and melanoma progression

This study was the first to report a coordinated decrease in mRNA expression levels of hKLKs (*KLK6, KLK7, KLK8 *and *KLK13*) as melanoma cells emerges from primary to metastatic phenotype, suggesting a role of these proteases in the epithelial-mesenchymal transition of primary melanoma cells. A recent study from our group evaluated the protein expression of hKLK6 and hKLK7 by a high-throughput Tissue Microarray Analysis (TMA) containing 162 independent PCM and 28 MM samples and showed augmented expression of these two hKLKs in PCM compared to MM [30]. The present study confirms these associations at the transcriptional level and shows that not only *KLK6 *and *KLK7 *but other hKLKs such as *KLK8 *and *KLK13 *are probably coordinately involved in melanoma progression. We also suggest that hKLK7 may be used as biomarker for melanoma progression, since increase in hKLK7 was significantly associated with good prognosis and survival (see additional file 4). Interestingly, it has been shown that hKLKs might also participate in early steps of neoplastic progression by directly or indirectly promoting or inhibiting cancer-cell proliferation [28].

**Figure 2:**
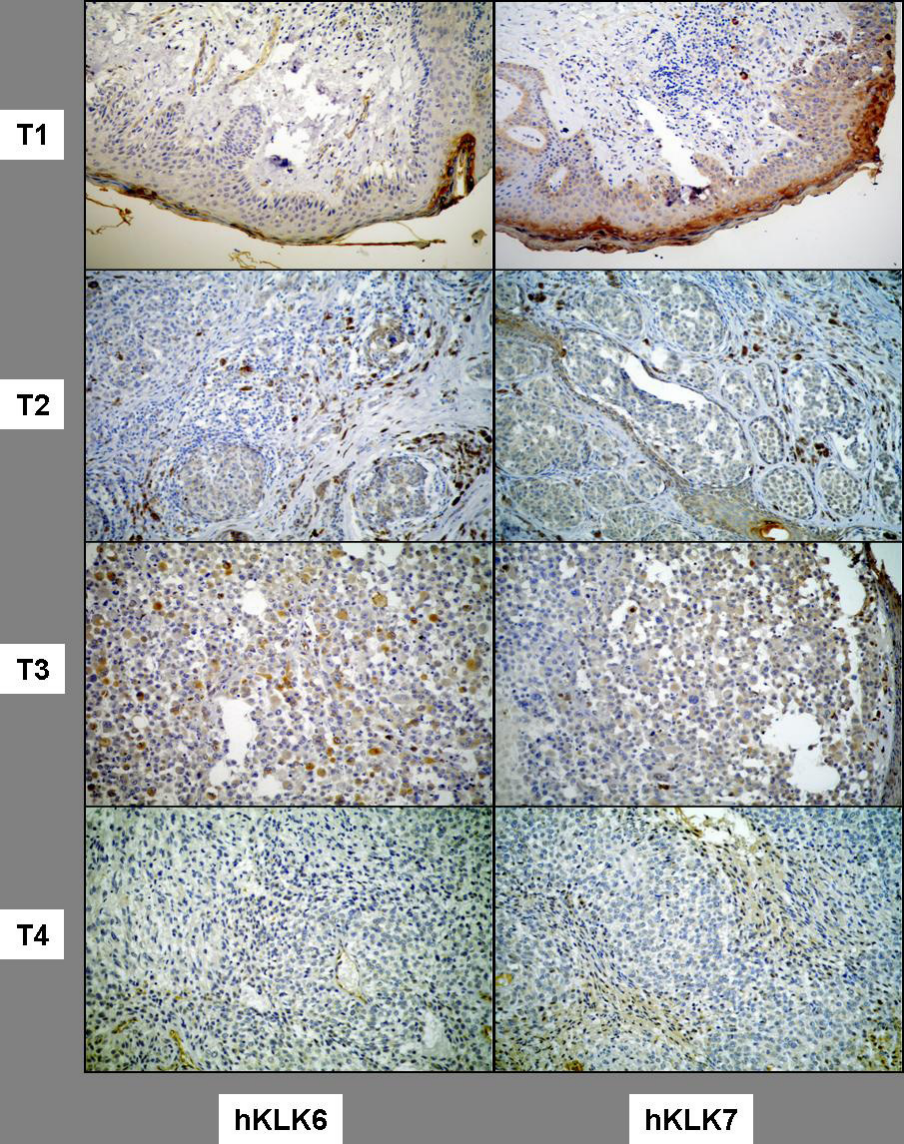
**Immunohistochemistry (tissue microarray - TMA) for hKLK7 and hKLK6 in four representative PCM samples with different Breslow's thickness index (T1 to T4)**. Original magnification 400×.

**Figure 3:**
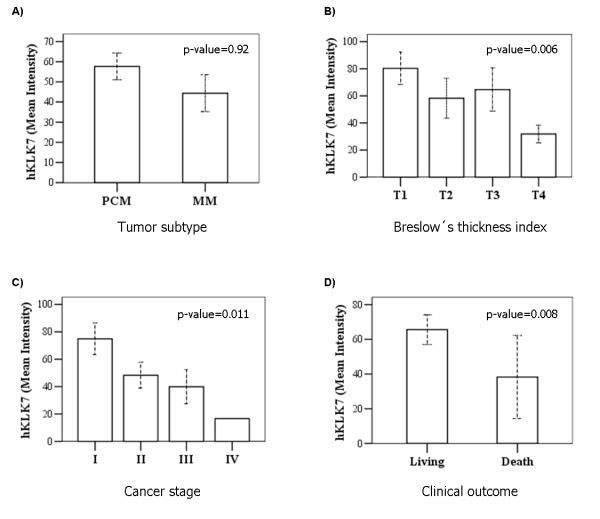
**Comparative statistical analysis of hKLK7 protein levels with respect to clinical and histopatological tumor characteristics**. Non-parametric Mann-Whitney and Kruskal-Wallis tests were used to compare median protein expression values from immunohistochemical quantitation done using ACIS III digital microscopy system with respect to Tumor subtype (A); Breslow's thickness index (B); Cancer stage (C) or Clinical outcome (D). Data derived from TMA assay (Figure 2). P-values lower than 0.05 were considered significant.

Figure 2 shows tissue microarray (TMA) analysis of hKLK6 and hKLK7 expression in four representative PCM samples with different Breslow's thickness index. As can be observed in the quantitative data shown in Figure 3, although higher levels of hKLK7 were found in patients with PCM in comparison to MM samples, the difference was not significant (p-value = 0.92). A decrease in hKLK7 expression was significantly associated with increase of Breslow's thickness index (p-value = 0.006), advanced disease (p-value = 0.011) and death by melanoma (p-value = 0.008). No association was observed between hKLK6 protein and tumor subtype or Breslow's thickness index.

**Figure 4:**
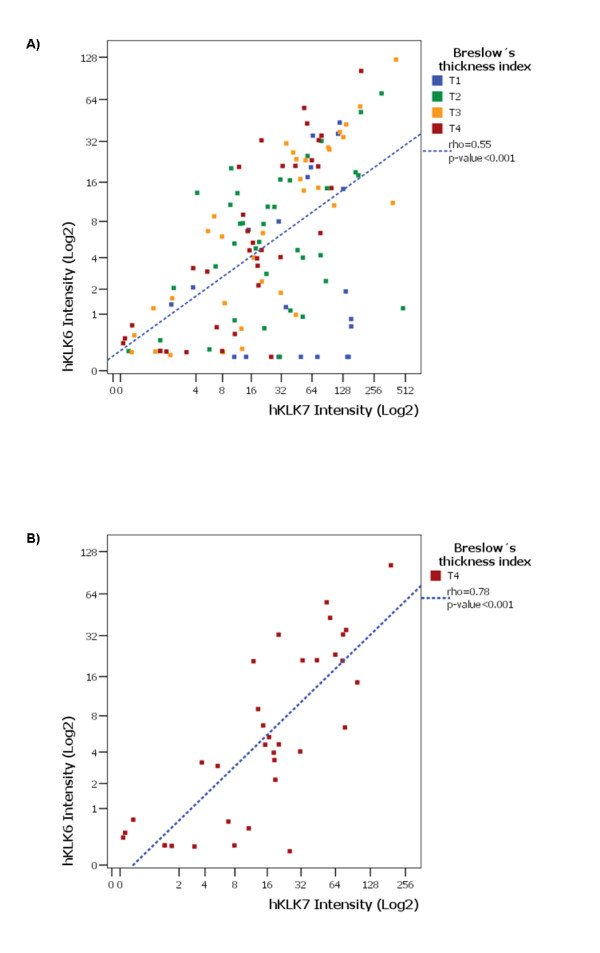
**Spearman's rho correlation analysis between expression levels of hKLK6 and hKLK7 in human PCM samples**. Scatter-plots showing a moderate Spearman's rho correlation coefficient (blue line) between the expression levels of hKLK6 and hKLK7 in PCM (0.3 to 19.5 mm in depth) samples (A), and in thick PCM samples (depth > 4.0 mm) (B).

Since we found a gene network correlating *KLK6 *and *KLK7 *mRNA expression (see additional file 3), we applied the linear Spearman's correlation coefficient (rho) to determine the degree of correlation between the expressions of the two genes at protein level measured by TMA. As can be observed in Figure 4, hKLK7 showed coordinated expression with hKLK6 with a regular Spearman's rho correlation coefficient (0.55, p-value < 0.001) in PCM samples, and moderate Spearman's rho correlation coefficient (0.78, p-value < 0.001) in thick PCM samples (depth > 4.0 mm). These data confirm the reliability of using gene networks to identify correlated gene expression. In contrast to the weak linear Pearson correlation coefficient (r = 0.083) between the expression levels (mRNA) of hKLK6 and hKLK7 in MM samples by our gene networks analyses, we also found a moderate correlation Spearman's rho (0.67, p-value < 0.001) between hKLK6 and hKLK7 protein levels in MM.

**Figure 5:**
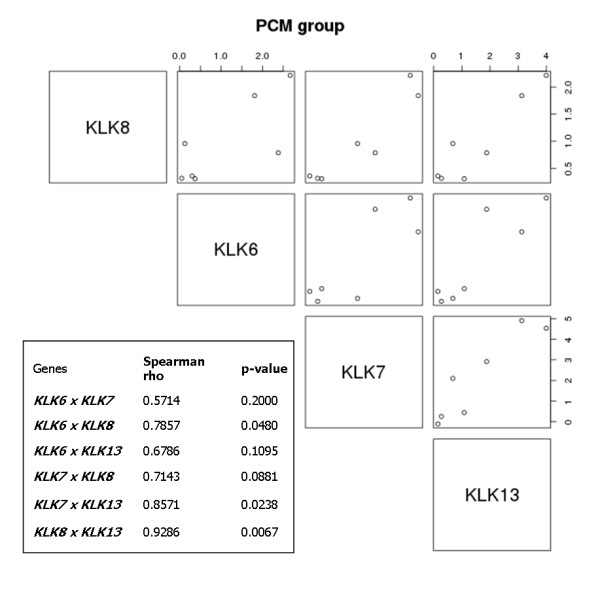
**Validation of the correlations among hKLK mRNA expression in an independent PCM dataset**. Scatter-plots showing Pearson's correlation coefficient between the mRNA expression levels of hKLKs in PCM samples. Each graph shows the expression of two kallikreins (the one in the left and the one below the graph) in all samples. The table shows correlation coefficient and the respective p-value for each kallikrein pair.

To confirm these observations in an independent group of samples we analyzed 7 PCM samples (additional file 5) using the same microarray platform, focusing in functionally hKLK-related genes network. The data presented in Figure 5 shows correlated expression not only between *KLK6 *and *KLK7 *but also between other *KLK *genes, reinforcing the association of hKLK module with primary melanoma progression.

## Discussion

Several expression studies have been performed in an attempt to identify genes that could characterize subtypes of human melanomas, as well as to define verifiable gene signature profiles with predictive value for metastasis, clinical outcome, or survival [9-19].

Although many differentially expressed genes have been found, there is still no generally accepted histopathological or molecular marker defining disease subsets with clinically different outcomes [11,15,16,54,55]. This frustrating situation reflects the complexity of working with melanoma samples and the difficulty of comparing data generated from different array platforms using distinct statistical analysis and diverse cohorts [9].

Thin melanomas are the most difficult samples to obtain in a research setting. For this reason, our thin PCM sample set is limited to a few samples collected during this study. Overall, the PCM set comprised mostly melanomas in the vertical growth phase with average PCM thicknesses of 3.18 mm and 10.82 mm for intermediate and thick tumors, respectively. Consequently, the genes identified here may be relevant for the transition from intermediate to thick tumors (and proceeding on to metastasis), but they are not especially informative for earlier steps in tumor progression. The similarity between the expression profiles of thick PCM and MM already described in previous studies [10] may explain why we could not precisely separate PCM from MM in our clustering SOM analysis (Figure 1).

To date, few studies have performed ontological comparisons of genes associated with specific biological processes [10,18], and none has analyzed coordinated gene expression. Here, we have investigated ontologically-linked gene sets with coordinated expression changes [51] that correlate with disease progression. We believe that changes in the relative expression of co-regulated genes that are ontologically-related may be more useful for predicting biological pathways in melanoma progression than the previously performed analysis of changes in single genes.

With this analytical approach we found a significant suppression of biological processes related to cell communication, cell adhesion, epidermal development, and epidermis cell-shedding as melanoma cells emerges from a primary to a metastatic phenotype (Table 1). This loss of cell-stroma cross-talk is a well-known behavior of melanoma cells that acquired an increased migratory potential in an epithelial-mesenchymal transition. These findings were concordant with a previous ontological analysis that identified reduced expression or loss of genes involved in keratinocyte differentiation, epidermal development, cell adhesion, and cell-cell signaling in metastatic samples [10,55].

We also observed changes in genes related to cell-to-cell and cell-to-matrix interactions that may modulate cross-talk between melanoma cells and the surrounding stroma: extracellular matrix structural components (*COL3A1, COL4A1, COL17A1, FBN1, FBLN1, FBLN5, LUM, LAMA3 *and *LAMA4*), adhesive molecules (*ALCAM, C1QR1, CYR61, DSC3, ITGB5, THBS1, THBS3, THBS4*), proteases involved in degradation of the extracellular matrix and possibly tumor cell migration and proliferation (*C1R, DPP4, KLK6, KLK7, KLK13, PLAU*), connective tissue factors (*CTGF, VWF*) and chemotactic factors (*CCL18, CCL20, CYR61*) that mediate cell attachment and tumor cell migration through cell-to-matrix interactions. Furthermore, we identified changes in *EMP1 *and *SLPI*, which play important roles in cell-cell adhesion, keratinocyte differentiation and cell shedding. Some of these genes had already been described in earlier studies as being down-regulated during melanoma progression: *COL17A1 *[15,18], *DSC3 *[15,18], *KLK7 *[10,15], *SLPI *and *KLK8 *[18], or over-expressed, e.g. *CCL20 *[55], *THBS1 *[55] and *THBS4 *[15]. However, other genes were here described for the first time as differentially expressed in melanoma tumors, such as *COL4A1, FBN1, LAMA3, LAMA4, C1QR1, CTGF, VWF, CTGF, PLAU*, *CYR61*, and *EMP1*.

Based on clustering data, we have identified a decreased expression of *KLK7 *in the PCM samples clustered with most MM samples. This sample set comprised thick melanomas (mean thickness 14.51 mm) that were significantly associated with poor prognosis (p-value = 0.003). Interestingly, changes in the *KLK6 *and *KLK7 *expression profile were associated with the transition from thin to thick cutaneous melanomas, being also significantly correlated to other genes functionally related to keratinocyte differentiation, cell shedding, invasion, and migration (Figure 1).

hKLKs have been implicated in carcinogenesis and often may serve as biomarkers for the screening, diagnosis, prognosis, and monitoring of certain prostate, ovarian, breast, testicular, lung and cervical cancers [22-29]. Accumulating evidences based on their epidermal localization and substrate specificity suggest that hKLKs (1, 4, 6, 8, 9, 10, 11, 13 and 14) are desquamation-related enzymes [33]. hKLK7 has been implicated in keratinocyte cell shedding, catalyzing the degradation of intercellular cohesive structures (corneodesmosomes) at the skin surface [32,34], an activity well controlled by specific protease inhibitors such as *SLPI *(secretory leukocyte protease inhibitor) [48]. Another important role ascribed to hKLKs is their involvement in extracellular matrix degradation. In this context, hKLKs 6, 7 and 13 have been implicated in the degradation of extracellular matrix proteins such as fibronectin, laminin, vitronectin and collagen [56-59]. Recently, hKLK8 was demonstrated as an active serine protease in human sweat and non-palmoplantar stratum corneum, also suggesting its involvement in skin desquamation and antimicrobial proteolytic cascades [60].

Our findings showed that not only the well studied hKLK7 but also other members of hKLKs-related genes were regulated during melanoma progression. In fact, we observed correlation at protein level between hKLK6 and 7 in PCM samples, and validated this observation by transcript analysis in an independent dataset.

We believe that even using a limited customized array platform that does not include all genes that may be involved in human carcinogenesis, we were able to add important information to melanoma progression due to the construction of a cancer related cDNA array and a careful selection of melanoma related modules.

The strategy of analysis of coordinated gene expression may help to identify pathways that act in concert to promote the complex process of melanoma development and metastasis. The precise mapping of these pathways may be very important before trying to interfere in tumor progression, since simultaneous genes should be targeted by therapy to reach a considerable benefit.

## Conclusions

In this study we analyzed coordinated gene expression changes to find molecular pathways involved in melanoma progression. We employed a different type of microarray analysis to evaluate the expression profile of 20 primary and 40 metastatic human melanomas. We identified ontologically-linked modules with coordinated expression changes in melanoma samples. We then detected several gene networks related to the different modules that were induced or repressed during melanoma progression. Among them we observed high coordinated expression levels of genes involved in cell communication, epidermal development and the functionally hKLK-related genes. Our findings showed that the well studied *KLK7 *and other hKLK members (*KLK6, 8 *and *13*) were coordinately expressed during melanoma progression: These findings were also validated in an independent dataset. Our data also indicated that hKLK7 protein expression was significantly associated with good prognosis and survival in PCM samples.

This study, employing a different type of analysis of microarray data, highlights the importance of analyzing coordinated gene expression to find molecular pathways involved in melanoma progression.

## List of abbreviations used in this paper

PCM: Primary cutaneous melanoma; MM: Metastatic melanoma; CS: Cancer Stage; LN: Lymph node metastases; CUT: Cutaneous metastases; VISC: Visceral metastases; T: Breslow's thickness index; S: Surviving; D: Death; AJCC: American Joint Committee on Cancer; hKLKs: Human tissue kallikreins.

## Competing interests

The authors declare that they have no competing interests.

## Authors' contributions

WKM conceived the study, participated in its design, and was involved in microarray studies, gene expression analysis and manuscript preparation. GHE was involved in microarray data analyses, statistical modeling, and manuscript preparation. OMA, GGR, GL and JPD were involved in the clinical and pathological characterization of melanoma samples. SMM and AFC supervised the cDNA microarray facility. LFLR participated in the study design and coordination. BSS participated in study design, analysis and manuscript preparation. All authors have read and approved the final version of the manuscript.

## Pre-publication history

The pre-publication history for this paper can be accessed here:

http://www.biomedcentral.com/1755-8794/4/76/prepub

## Supplementary Material

Additional file 1 **Description of PCM and MM samples**. Samples are described with respect to tumor histopathological and clinical characteristics: tumor subtype, Breslow's thickness index (mm), TNM, pathologic stage, patient status, follow up. Click here for file

Additional file 2 **Genes that contributed most significantly for the 22 altered modules shown in **table 1. List of genes that most contributed for the non-redundant modules identified in this work. This file describes gene names, scores and p-values for each module in distinct melanoma categories: tumor subtype, melanoma progression, cancer stage, metastasis site and clinical outcome. Click here for file

Additional file 3 **Gene networks with high correlation coefficients in human melanoma samples**. List of the pairs of genes with highest correlation significance (cut off set as 10^-4.3^) in the three most relevant altered modules: cell communication (GD), epidermal development (GO8544), functionally hKLK-related. This file describes the correlation coefficients and the significance (p-values) for each pair in distinct categories: tumor subtype, cancer stage and melanoma progression. Click here for file

Additional file 4 **Statistical analysis of hKLK6 and 7 protein expression according to clinical and histopathological parameters**. Immunohistochemistry data analyzed by non-parametric tests (Mann-Whittney and Kruskal-Wallis) according to clinical outcome, ulceration, Breslow's thickness index and cancer stage. Click here for file

Additional file 5 **Description of PCM samples from an independent microarray dataset**. PCM samples are described with respect to tumor histopathological and clinical characteristics: Breslow's thickness index (mm), TNM and pathologic stage. Click here for file
